# Design and Evaluation of an Adjustable Compliant Constant-Force Microgripper

**DOI:** 10.3390/mi15010052

**Published:** 2023-12-26

**Authors:** Jiahang He, Yinong Liu, Chunbiao Yang, Zongdi Tong, Guangwei Wang

**Affiliations:** School of Mechanical Engineering, Guizhou University, Guiyang 550025, China

**Keywords:** compliant mechanism, constant-force mechanism, microgripper, adjustable constant force

## Abstract

Precise control of the manipulating force within an appropriate range is crucial to prevent potential damage to the operating object. However, achieving accurate force control through force feedback is challenging in micro-scale applications. This study presents the design of a quasi-zero stiffness-compliant constant-force microgripper with adjustable force output. The parameters of the constant-force mechanism are designed using a model-based optimization method. By utilizing this mechanism, a compliant microgripper capable of providing adjustable constant-force output is developed to overcome the limitation of traditional grippers that offer only a single constant force. Finite element analysis is performed to simulate the behavior and verify the stability of the constant-force output. Furthermore, an experimental platform is constructed to validate the mechanical properties of the developed microgripper. The experimental results demonstrate that the automatically optimized structural parameters enable the microgripper to achieve the desired constant-force value of 2 N with an adjustable range of 0.15 N. These findings provide a further basis for the application and promotion of compliant constant-force structures.

## 1. Introduction

With the continuous advancements in automation technology, micro-electrical mechanical systems, and intelligent manufacturing, industrial production automation and intelligence have become the prevailing trends. In order to cater to diverse requirements, automation equipment must be equipped with suitable end-effectors tailored to specific usage scenarios. Grippers, as typical end-effectors of mechanical arms, have found wide applications in the industrial sector. Grippers used in large-scale automated production lines are typically designed with high stiffness and a wide clamping range to accommodate various handling, assembly, and positioning tasks. However, in fields such as precision operation technology [1], micro-assembly [2], bioengineering technology [3], micro-electrical mechanical operation [4], and polishing grinding technology [5], precision components, biological cells, and other delicate objects exhibit notable fragility and deformability. These objects are prone to damage during the gripping process. To mitigate such risks, microgrippers must achieve precise operating force control by controlling the position within an appropriate range [6].

The quasi-zero stiffness-compliant constant-force mechanism (CFM) offers an alternative to complex force control systems, overcoming the challenges related to poor sensor anti-interference ability and complex system integration [7,8]. Compared to a traditional rigid gripper, the compliant constant-force gripper eliminates the influence of friction wear and clearance, thus improving accuracy. Additionally, it simplifies the complex force/position cooperative control problem into a simpler single-position control problem, reducing the controller design [9]. It utilizes the deformation of its compliant components to realize force transmission and can provide nearly constant force within a specific range [10]. In most cases, constant-force mechanisms achieve quasi-zero stiffness through coupled buckling deformation, resulting in a consistent output force [11]. This characteristic renders it particularly suitable for high-precision applications, which hold significant value for advancing precision operation and micro-manufacturing industries.

The current types of CFM are primarily classified based on principles such as Stiffness Combination Constant-force Mechanism (SCCFM), Curved Beam Constant-Force Mechanism (CBCFM), Shape Optimization Constant-Force Mechanism (SOCFM), Cross Spring Constant-Force Mechanism (CSCFM), and Constant-Force Compression Mechanism (CFCM) [12]. The main theoretical modeling methods used in CFM research include the elliptic integral method, finite element method, pseudo-rigid body model method, and chain algorithm [13,14,15]. Moreover, to accommodate different operating force requirements of target objects, numerous studies have been conducted on the adjustable constant-force mechanism and multi-stage constant-force mechanism. Chen et al. proposed the concept of an adjustable constant-force mechanism (ACFM) and designed a single ACFM [16]. The principle is adjusting the constant-force output by applying a preloading displacement to the constant-force mechanism. Kuo et al. designed a Two-Dimensional ACFM using the same theory [17]. Another approach to achieving multi-stage constant-force output is through the assembly or disassembly of different-sized constant-force mechanisms, as seen in the research content in [18,19,20]. While previous studies have attempted to address the limitations of constant-force mechanisms by introducing multi-stage constant-force mechanisms and adjustable constant-force mechanisms, these solutions often suffer from the inability to customize the output force and the lack of compactness in their structure. These factors make them unsuitable for precision operation scenarios.

During the design process of CFMs, solving the inversion equations of bistable beams proves to be complex. As a result, the current design process of constant-force mechanisms heavily relies on trial and error until the appropriate structural parameters are determined. However, manually finding the optimal solution is challenging and time-consuming due to the multi-objective nature of the optimization problem. To overcome this challenge, the multi-objective genetic algorithm (MOGA) has been developed as an effective approach to address global optimal problems in engineering fields [21]. Moreover, in order to achieve multi-stage or stepless adjustable constant-force mechanisms, a potential solution lies in utilizing PZT-driven compliant mechanisms. PZT has been widely used in precise manipulation scenarios due to its characteristics of large force, high resolution, and fast response [22,23]. Since the displacement output of PZT is small, it often needs to be used with an amplification mechanism [24]. Among these mechanisms, the bridge-type amplifier has been widely employed due to its advantages of large magnification and compact size [25].

The main contribution of this paper is the design of a compliant constant-force microgripper with adjustable output, incorporating automatic optimization of constant force and adjustable force output functionality. Specifically, based on the proposed design framework, the only requirement is to design a preliminary mechanism that approximates the output with the desired constant-force value. Through automatic optimization, these parameters can be adjusted to achieve the desired constant-force value while also extending the range of constant-force output. This approach greatly reduces the design difficulty and time consumption in the conventional design process. In addition, by utilizing the designed piezo-driven adjustable module, the microgripper enables stepless adjustment of the constant-force output, which allows for a broader range of applications to be accommodated.

The rest of this paper is organized as follows: Section 2 introduces the theoretical model of the bistable beam and the positive stiffness beam. Section 3 outlines the process of the automatic optimization framework for the complaint constant-force mechanism and the design of the microgripper with a stepless adjustable constant-force function. The performance simulation and testing of the developed microgripper are presented in Section 4. Finally, the conclusion and the summary are drawn in Section 5.

## 2. Theoretical Model

**Figure 1:**
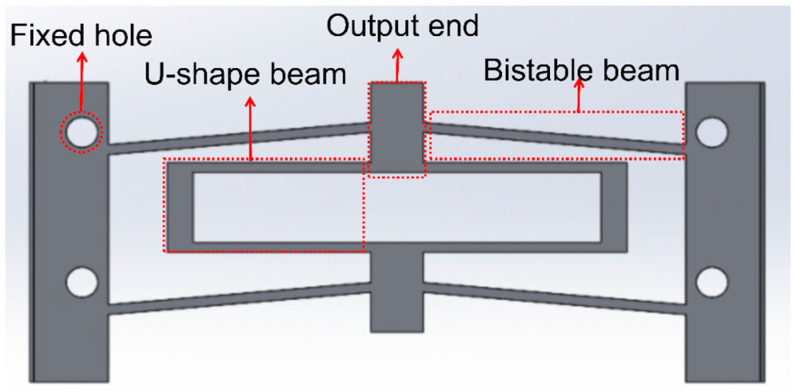
Structure of compliant constant-force mechanism.

The stiffness combination constant-force mechanism is utilized in this work due to its inherent advantages of simple structure, easy design, and high compactness [12]. In scenarios involving substantial deformations and intricate load conditions, the elliptic integral method proves more adept than other methods. A theoretical model of a bistable beam with negative stiffness is established by using the elliptic integral method. Concurrently, the pseudo-rigid body model is utilized to establish the theoretical model for the U-beam with positive stiffness. By integrating these two theoretical models, the theoretical model for the quasi-zero stiffness-compliant mechanism is derived. Figure 1 shows the structure of the compliant constant-force mechanism to illustrate the principle of stiffness combination. This mechanism consists of two pairs of symmetrically distributed bistable beams positioned above and below a pair of U-shaped beams. The U-shaped beams contribute positive stiffness to the overall mechanism, while the bistable beams offer negative stiffness within a specific range through compliant coupling. When the output end makes contact with an external object, the bistable beams deform to provide negative stiffness. This negative stiffness is then combined with the positive stiffness provided by the U-shaped beams, resulting in zero stiffness at the appropriate moment. Consequently, the mechanism achieves the desired characteristic of a compliant constant force. The specific theoretical model is introduced in the following section.

### 2.1. Theoretical Model of Negative Stiffness Mechanism

**Figure 2:**
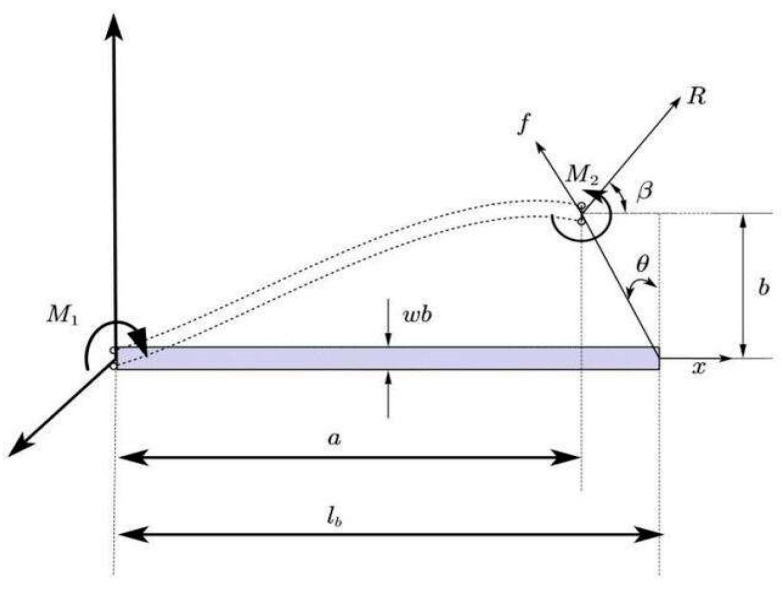
Structural parameters of bistable beam.

In the process of bending deformation, the manifestation of a buckling effect imparts bistable characteristics, resulting in the generation of a negative stiffness range [20,26]. According to the analysis of Figure 2, the moment of a specific point on the beam can be mathematically expressed using the Euler–Bernoulli equation as follows:

(1) $$EI\frac{d(\beta -\theta )}{ds}=M+Rasin\beta +Rbcos\beta$$

where E represents the Young’s modulus of the beam; $I=\frac{\omega_{b}{t_{b}}^{3}}{12}$ represents the moment of inertia of the beam, which is defined based on the assumption that the beam has a rectangular section; $t_{b}{,\;\omega }_{b}$, and θ represents the thickness, width, and inclination angle of the bistable beam; β represents the force angle with respect to the X-axis; ds represents the differential length of the bistable beam $l_{b}$; and a and b represent the horizontal coordinate and ordinate of any point on the beam. M and R represent the torque and force applied to the beam, respectively.

According to [27,28,29], this paper omits the theoretical derivations involving integrals, differentials, and trigonometric conversions, opting instead to directly utilize the outcomes derived from the bistable beam mechanical model. The total driving force of the negative stiffness mechanism is expressed as

(2) f=R (cosβsinθ+sinβcosθ)

### 2.2. Theoretical Model of Positive Stiffness Mechanism

**Figure 3:**
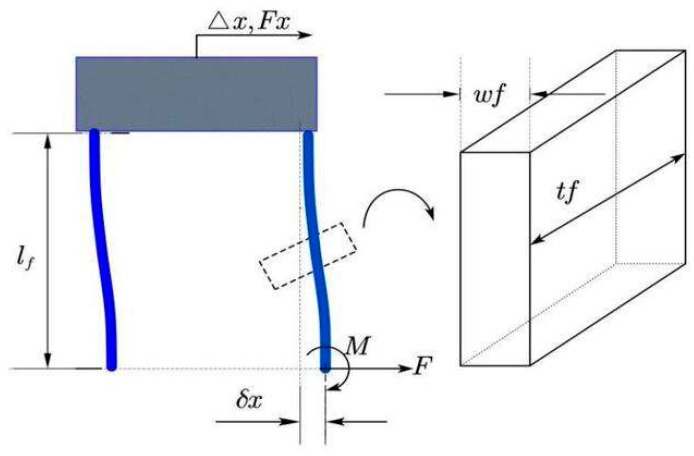
Structural parameters of U-shaped beam.

The U-shaped beam composed of compliant structures serves as the positive stiffness mechanism in this work. Due to the symmetrical distribution and identical dimensions of the U-shaped mechanisms, the fixed guide beams experience uniform deformation [30]. The equivalent stiffness of the positive stiffness mechanism, as deduced in [31] and shown in Figure 3, can be expressed as follows:

(3) $$K_{f}=\frac{F_{x}}{∆x}=\frac{F}{2\delta_{x}}=\frac{{Et}_{f}{\omega_{f}}^{3}}{{l_{f}}^{3}}$$

where E is the Young’s modulus of the material, and $t_{f,}{\;\omega }_{f,\;\;}\;l_{f}$ represent the thickness, width, and length of the beam, respectively.

### 2.3. Theoretical Model of Compliant-Force Mechanism

Upon obtaining the mechanical models for the positive and negative stiffness mechanisms, the relationship between structural parameters and their influence on the output force is delineated in this subsection. Based on the principle of positive and negative stiffness combination, the theoretical output force of the quasi-zero, stiffness-compliant, constant-force mechanism is as follows:

(4) $$F_{z}=2f+F$$

where $F_{z}$ represents the total driving force of the quasi-zero stiffness-compliant mechanism, f represents the driving force of the individual negative stiffness mechanism, and F represents the driving force of the positive stiffness mechanism. Substituting (2) and (3) into (4) yields

(5) $$F_{z}=2R(cos\beta sin\theta +sin\beta cos\theta )+\frac{{Et}_{f}{\omega_{f}}^{3}}{{l_{f}}^{3}}x$$

## 3. Design of Microgripper

Due to the complexity of equation inversion to solve bistable beams, the current design process of constant-force mechanisms heavily relies on trial and error to determine the appropriate structural parameters based on the previous mathematical model. This poses a significant challenge as it involves a multi-objective optimization problem, and manually finding the optimal solution can be impossible and time-consuming. This study introduces an automatic optimization framework based on FEA results and the MOGA method. The framework can be delineated into three steps: (1) establishing the preliminary model with coarse parameters, (2) conducting automatic optimization of model parameters, and (3) undertaking the structure design of the adjustable constant-force microgripper.

### 3.1. The Establishment of the Preliminary Model

#### 3.1.1. Parameter Selection of the Preliminary Model

Building upon the theoretical modeling of the compliant constant-force mechanism, a parameter set for the preliminary model can be quickly determined. This preliminary model can be roughly determined through a trial-and-error process, with the aim of achieving a constant-force output in the vicinity of the predefined value. A simple example of such a parameter set is shown in Table 1.

#### 3.1.2. Simulation and Test of the Preliminary Model

**Figure 4:**
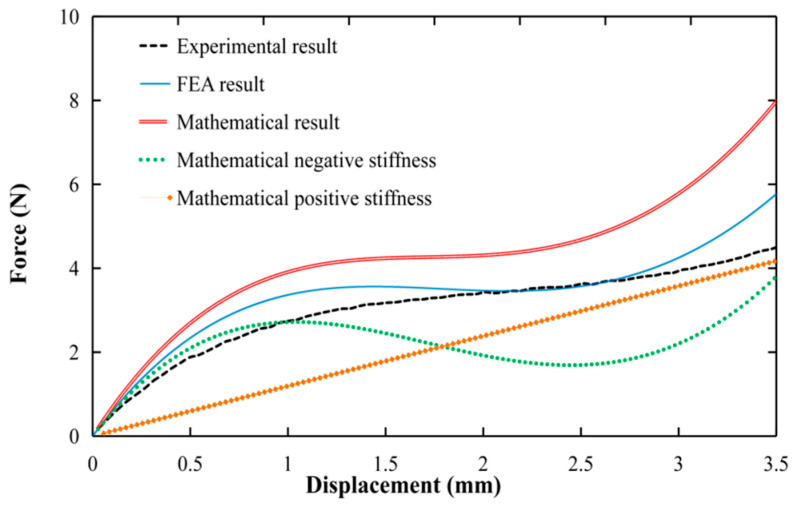
Comparison of mathematical results, FEA results, and experimental results of the preliminary constant-force mechanism.

To verify the accuracy of the mathematical model and assess its suitability for model optimization, this section includes numerical analysis, FEA simulation, and experimental validation using the preliminary model parameters outlined in Table 1 and the structure depicted in Figure 1. The ABS plastic material is adopted for the FEA and FDM 3D printing prototype; its mechanical properties are provided in Table 2, the FEA results are obtained using ANSYS Workbench (2021 R1) static structure analysis, the experimental setup aligns with the depictions in Figures 10 and 14, and the detailed description can be found in Section 4. The comparative analysis results are shown in Figure 4.

It is evident that a significant performance discrepancy exists between the mathematical model and the experimental results. However, the constant force obtained from FEA is found to be more consistent with the experimental results, exhibiting a 17.8% reduction compared to the mathematical model. This finding indicates that direct optimization based on the mathematical model did not yield effective structural parameters and that FEA provides a more accurate representation of real-world conditions. Hence, this paper proposes a two-step approach for optimizing constant-force mechanisms: firstly, the mathematical model is used to quickly obtain a set of structural parameters that are moderately close to the desired performance, and second, automatic optimization is performed based on the FEA results to obtain mechanism parameters that are more aligned with the actual system.

### 3.2. Automatic Optimization of Constant-Force Mechanism

#### 3.2.1. Optimization Objective

The multi-objective genetic algorithm possesses the advantage of effectively identifying the global optimal solution. In this study, the design objective of the quasi-zero stiffness-compliant mechanism is to achieve a constant-force output while maximizing the constant-force stroke. Consequently, the optimization problem can be formulated as described in [25].

(6) $$To:\left\{\begin{array}{c} F_{c}(\omega ,t,\theta ,l_{f})=F_{d}\\ max{\;S}_{c}(\omega ,t,\theta ,l_{f}) \end{array}\right.$$

where $F_{C}$ is the output of CFM; $S_{C}$ represents the constant-force range; $F_{d}$ is the desired constant-force output; ω,t,θ, and $l_{f}$ are the structural parameters to be optimized; and t and ω indicate the thickness and width of the compliant beams, respectively.

#### 3.2.2. Constant-Force Performance Evaluation

**Figure 5:**
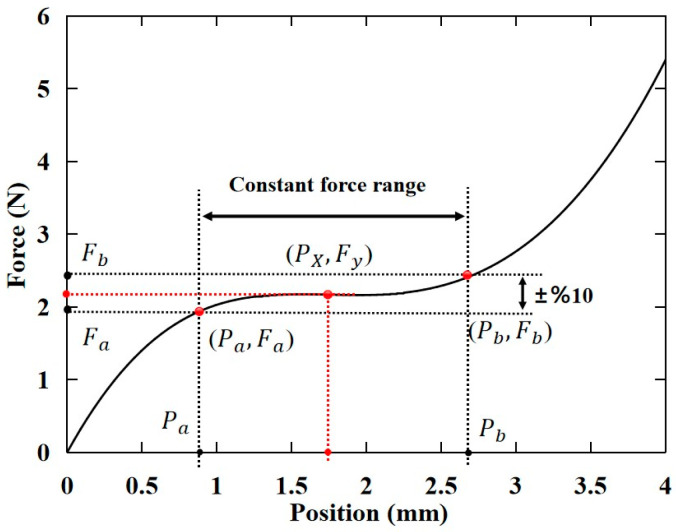
Schematic diagram of the sampling point.

To establish the optimization evaluation index, a sampling point ($P_{x}{,F}_{y}$) is selected from the force-displacement curve, as illustrated in Figure 5. $P_{x}$ represents the displacement input, and $F_{y}$ is the output force at the current displacement point. n denotes the number of random points selected in the optimization process (n ≧ 3). In addition, the slope $k_{n}$ between two selected points is introduced as a criterion for identifying the quasi-zero stiffness stroke. If the slope between the selected points consistently approaches zero, it ensures that all selected random points are within a constant-force range.

Moreover, combined with the actual working situation, the average value of total deformation (TDA) and the maximum value of equivalent stress (ESM) are employed as auxiliary judgment criteria. A large TDA and a small ESM indicate high deformation resistance and a more compact structure. To bring the convergence results closer to reality, the largest TDA and the smallest ESM are typically chosen.

Based on the defined content, the cost function that incorporates multiple evaluation metrics for the constant-force mechanism can be formulated as follows:

(7) $$min\;\mu =p\left(\left|F_{d}{-F}_{y}\right|\right)-q\left(P_{b}-P_{a}\right)+\theta \sum_{n=1}^{3}k_{n}-\sigma TDA+\varphi ESM$$

where μ is the cost function, which is used to explain Equation (6). To meet the requirements of Equation (6), it is necessary to find the minimum value of the cost function. p,  q,  θ,  σ, and φ are weight coefficients whose sum is 1; $F_{y}$ represents force; $P_{x}$ represents displacement; $k_{n}$ is slope; TDA is the overall deformation average; and ESM is the maximum equivalent stress.

#### 3.2.3. Automatic Parameter Optimization

**Figure 6:**
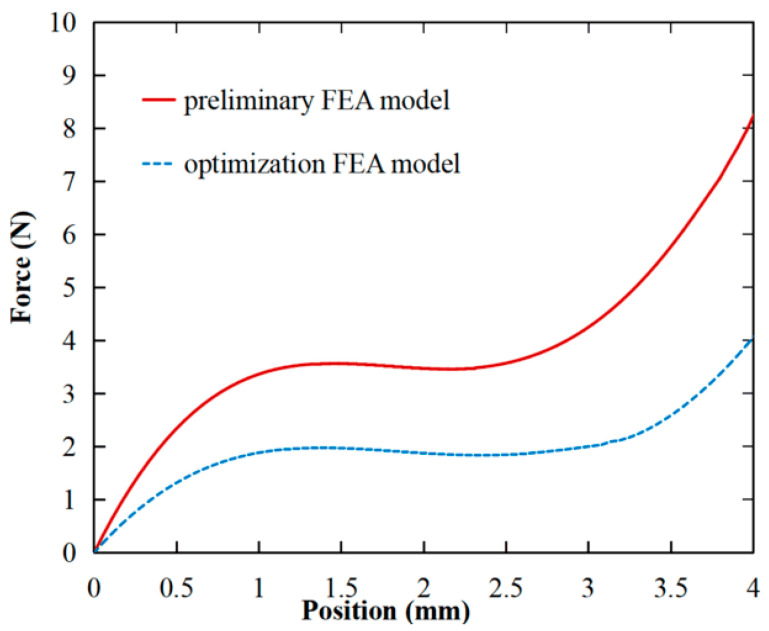
Comparison diagram between the preliminary FEA model and the optimized FEA model.

The objective function optimization criteria in (7) are employed on the MOGA algorithm in ANSYS software, with the following specifications: the desired constant-force output is $F_{d}$ = 2 N, the number of sampling points is *n* = 3, and the optimization precision for constant force is ±10%. The final values of the optimized structure parameters are shown in Table 3. The simulation results of the force–displacement curves corresponding to the optimized model parameters are shown in Figure 6.

It can be observed that the preliminary model, with a coarse parameter setting, resulted in a constant-force output of 3.50 N and a corresponding constant-force stroke of 1.87 mm, ranging from 0.88 to 2.75 mm. In contrast, the optimized model exhibited a constant-force output of 1.90 N, with a constant-force stroke of 2.26 mm, ranging from 0.82 to 3.08 mm. The performance error of the optimized FEA model, relative to the design target constant-force output of 2 N, is 5%. Additionally, the constant-force stroke increased by 20.9% after model optimization.

The advantages and novelty of the optimized model lie in its ability to automatically optimize the preliminary model to achieve the desired performance while achieving a large constant-force stroke. This approach effectively overcomes the time-consuming nature of conventional compliant constant-force mechanism design processes and addresses the challenges associated with multi-objective optimization.

### 3.3. Design of Adjustable Compliant Constant-Force Microgripper

**Figure 7:**
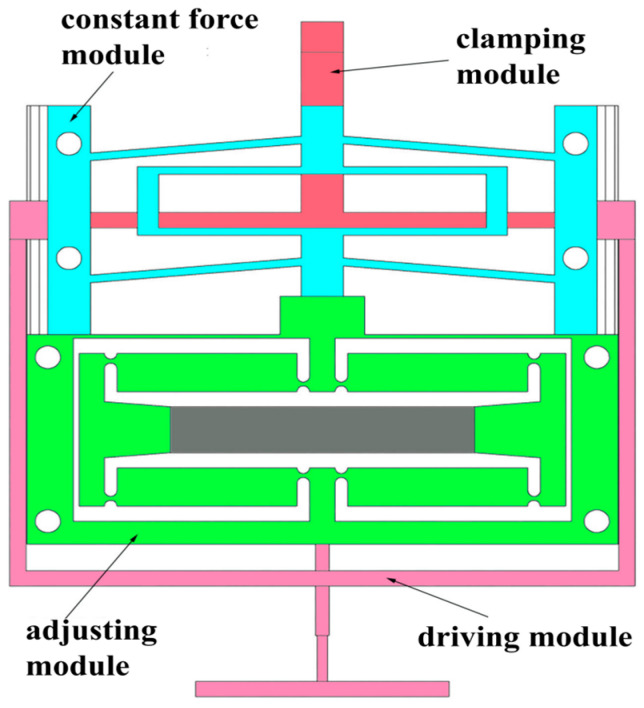
The overall design diagram of the quasi-zero stiffness-compliant microgripper.

The compliant constant-force microgripper with adjustable output is developed with four main modules: the driving module, clamping module, constant-force module, and adjusting module; its CAD model can be found in Figure 7.

The working principle is as follows: the optimized constant-force mechanism operates as the constant-force module, delivering a consistent clamping force output within the constant-force stroke. One end of this module is connected to the adjusting module, while the other end, together with the end of the clamping module, forms the jaws of the microgripper. The clamping module, functioning as a dynamic gripper mechanism, is interconnected with the driving module. The driving module can be actuated either manually or by a voice-coil motor. As the driving module moves, it induces the contraction of the clamping module, leading the gripper to close and thereby clamping the target along with the constant-force module. The adjusting module, situated between the driving module and the constant-force module, provides the necessary adjustment output. The adjusting module is designed based on a bridge-type amplifier driven by a piezoelectric actuator.

## 4. Simulation and Experimental Verification

This section includes both FEA simulation and experimental verification to evaluate the performance of the developed adjustable constant-force microgripper. Because the clamping module and the driving module have little effect on the performance of the microgripper, in order to simplify the difficulty of simulation and experiment, only the constant-force module and the adjusting module are selected for simulation and experiment.

### 4.1. FEA Simulation

**Figure 8:**
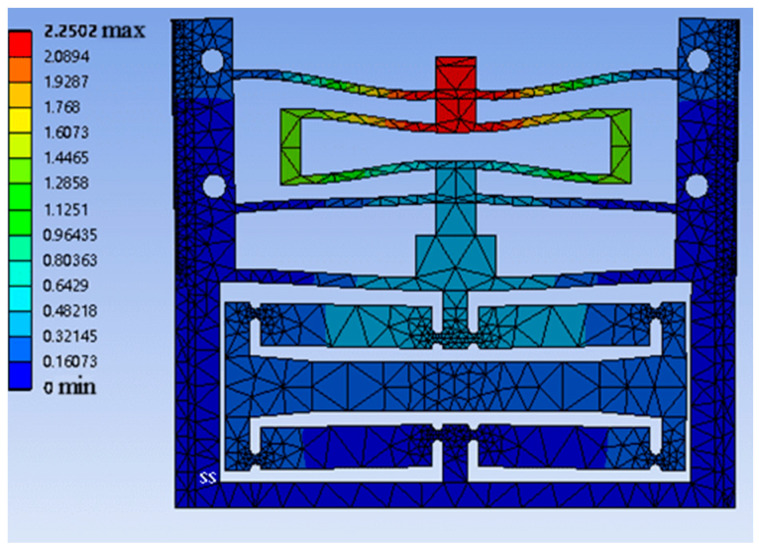
Overall deformation diagram of the microgripper model.

To validate the mechanical properties of the microgripper, the software ANSYS Workbench is utilized for the static structural analysis. ABS plastic is used as the microgripper material, and the mechanical parameters of the material can be found in Table 1. The analysis considers the large deformation of the mechanism while neglecting the shear deformation effect. In the simulation, the mesh size of 0.5 mm is used with tetrahedral cells and linear interpolation. It is important to note that the convergence results are not significantly affected by the mesh size. The FEA simulation is conducted on a host computer with an Intel(R) Xeon(R) CPU E5-2673 v4 @ 2.30 GHz and 64 GB RAM. To apply constraints, four fixed holes on the constant-force module are used. Displacement inputs are applied from top to bottom on the output end of the constant-force module. The overall deformation diagram is presented in Figure 8. The simulation results of the force–displacement characteristic curve of the microgripper can be observed in Figure 15.

**Figure 9:**
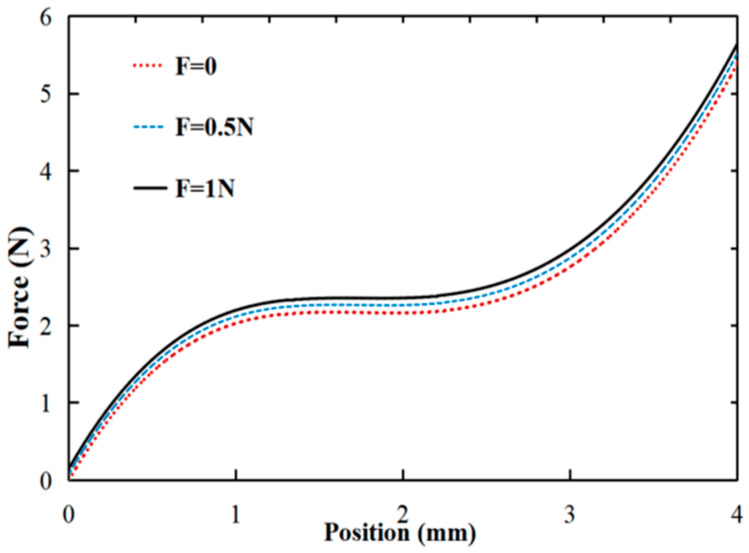
Simulation results of output force of microgripper under different preloading forces.

Simultaneously, FEA was conducted on the force–displacement curve of the microgripper under different preload conditions of the adjusting modules. The meshing method and material settings remained the same as described earlier. However, additional constraint conditions were applied to simulate the driving forces from 0 N to 1 N acting on the side wall of the PZT mounting hole in the regulation module. This analysis aimed to examine the effect of these forces on the output force. The results of this analysis can be observed in Figure 9.

### 4.2. Experimental Verification

#### 4.2.1. Experimental Setup

**Figure 10:**
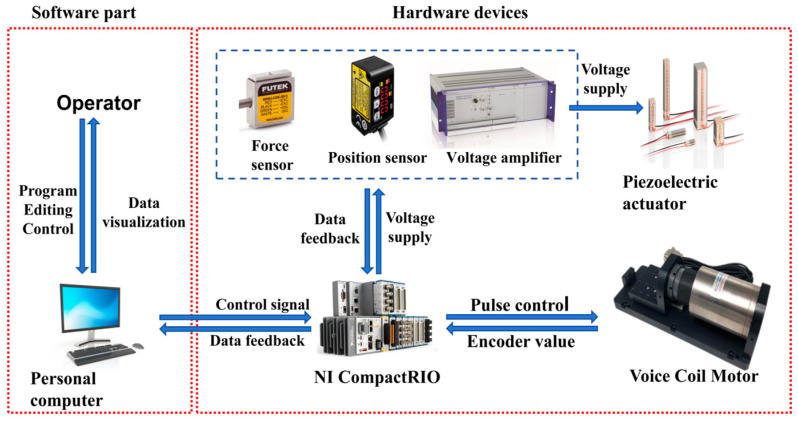
Frame of experimental platform.

The NI-Compact RIO (NI-9257, from National Instruments Corp., Austin, TX, USA) along with other digital IO expansion modules, are utilized to establish a connection between the host computer and the developed hardware platform. A force transducer (model: LSB201, from FUTEK Advanced Sensor Technology, Inc., Irvine, CA, USA) is employed to measure contact force in real-time. The position sensor (model: HGC-1030, from Panasonic Industry Co., Ltd., Osaka, Japan) with a range of ±5 mm and an accuracy of 10 μm is used to measure the displacement. The adjusting module is driven by the PZT (model: P-885.91, from Physik Instrumente, Co., Lederhose, Germany), capable of providing driving force, while the driving module is actuated by a voice coil motor. Additionally, a voltage amplifier (model: PI E-505.00S, from Physik Instrumente, Co., Lederhose, Germany) with a conversion constant of 10 is used to drive the PZT by amplifying the control voltage. The experimental setup framework is shown in Figure 10.

#### 4.2.2. Mechanical Properties of Adjusting Module

**Figure 11:**
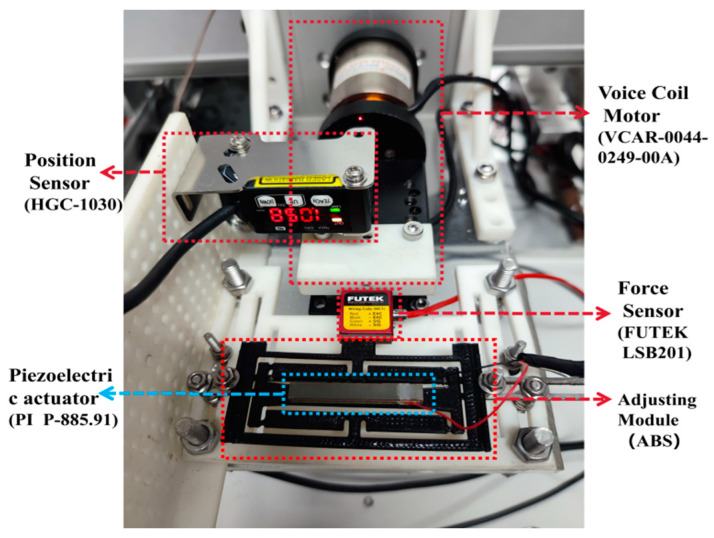
Testing platform for adjusting module.

To achieve precise adjustment of the output force of the developed microgripper, it is required to obtain the relationship between the driving voltage of the PZT and the output of the adjusting module. For this purpose, a testing platform was specifically constructed to facilitate calibration and performance testing of the adjusting module, as illustrated in Figure 11.

The experimental process involves utilizing a voice coil motor equipped with a force sensor to drive the microgripper. The force sensor is in direct contact with the adjusting module, simulating the preload output to the constant-force module under the working conditions of the microgripper. Since the designed constant-force output is set at 2 N, the position of the voice coil motor is adjusted before the experiment to ensure the desired preload force on the adjusting module. Under these initial conditions, the driving voltage of PZT is adjusted, and the output force and displacement of the adjusting module are measured.

**Figure 12:**
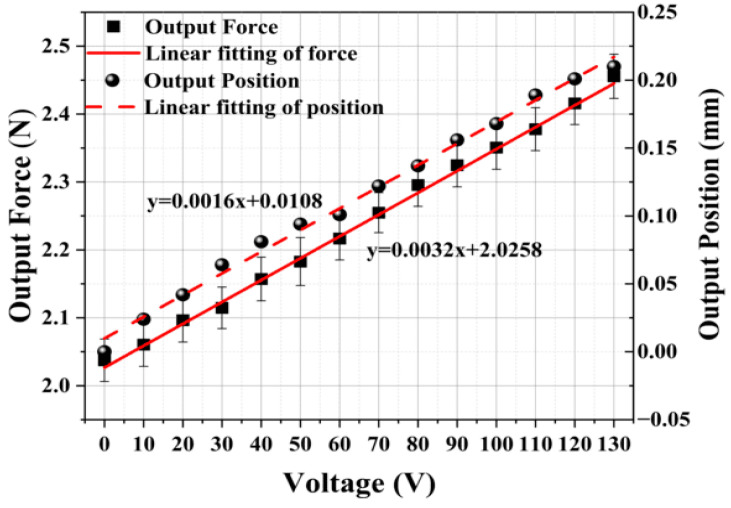
Output position and output force of the adjusting module.

**Figure 13:**
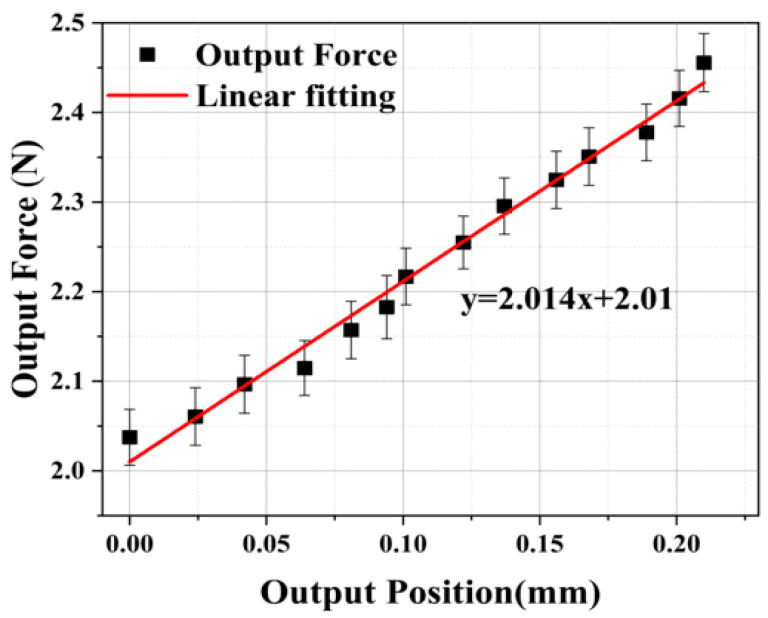
The relationship between the output force and the output position.

The experimental results are presented in Figure 12 and Figure 13. As the driving voltage of PZT gradually increases from 0 to 130 V, the output position of the adjusting module also gradually increases from 0 to 0.21 mm. Simultaneously, the output force increases from 0 to 0.45 N. The relationship between the output force and position is linearized and depicted in Figure 13. The test results of the adjusting module effectively calibrate and establish a unified relationship between the driving voltage of the PZT, output position, and output force. This relationship serves as the foundation for accurately adjusting the constant-force output of the microgripper. By knowing one of the driving voltages, regulating positions, or regulating forces, the magnitude of the other two values can be determined.

#### 4.2.3. Mechanical Properties of Microgripper

**Figure 14:**
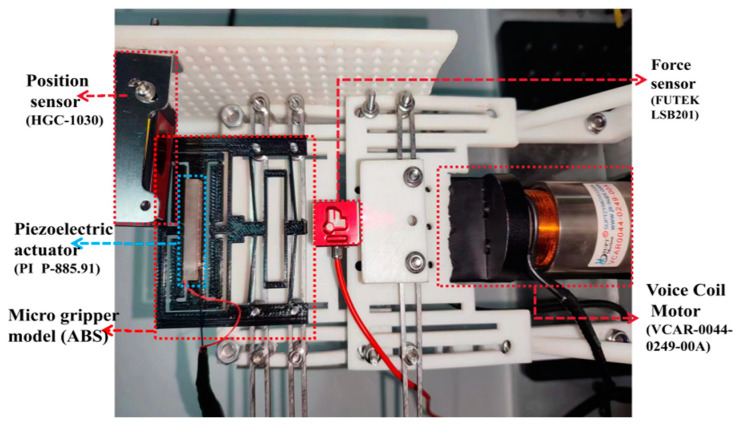
Testing platform for constant-force microgripper.

The experimental platform shown in Figure 14 is utilized to test the constant-force characteristics and adjustable performance of the microgripper. This platform consists of a force sensor mounted on the voice coil motor, allowing for synchronous movement. The microgripper is positioned on the baseplate at the same height as the force sensor to ensure proper alignment with the output end of the microgripper. The contact between the force sensor and the microgripper’s output end simulates the clamping effect.

The PZT is appropriately placed on the designated installation port of the microgripper. The force and displacement data from the microgripper are accurately collected using force and position sensors. The feedback data are acquired through the NI-Compact RIO module. The collected data are then transmitted to the host computer for effective visualization and post-processing. Voltage control signals are also sent to the voltage amplifier to precisely regulate the output force and displacement of the adjusting module.

**Figure 15:**
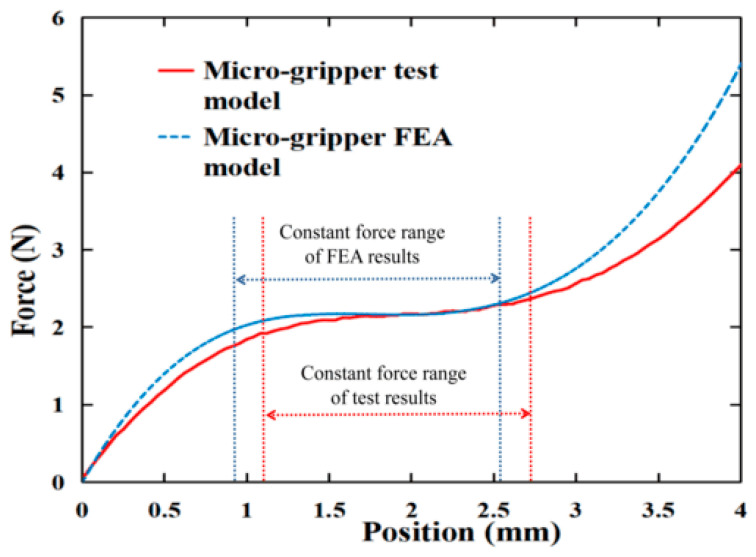
Comparison of simulation results and experimental results.

The constant-force output characteristics of the microgripper are evaluated using the experimental platform, and a comparison is made with the results obtained from FEA, as shown in Figure 15. The experimental results of the microgripper model indicate a constant force of approximately 2.1 N; the constant-force range is measured to be between 1.125 mm and 2.722 mm, resulting in a total constant-force range of 1.59 mm. The FEA results of the microgripper exhibit a constant-force output of around 2.1 N, with a constant-force range spanning from 0.855 mm to 2.565 mm, resulting in a constant-force range of 1.71 mm.

Although the experimental and simulation results show similar constant-force outputs, there are some discrepancies in the constant-force intervals. There might be several potential factors contributing to this discrepancy. One possible reason is the inherent error between FEA and experimental results of the constant-force mechanism, as shown in Figure 4. As the structure of the microgripper becomes more complex, the inherent error gradually accumulates. Another potential reason for the discrepancy is the manufacturing error. To facilitate quick testing, the prototype of the developed microgripper is manufactured using 3D printing. The accuracy of the compliant hinge fabrication significantly affects the performance of the constant-force mechanism. However, limitations in the accuracy of 3D printing and errors in the assembly process can also lead to differences between the final test results and simulation results. Additionally, deviations in the experimental results can also arise from sensor errors. However, it is worth noting that the experimental results are generally consistent with the simulation results. The error in the constant-force interval is calculated to be approximately 7%, indicating a reasonable level of agreement between the experimental and simulated results.

**Figure 16:**
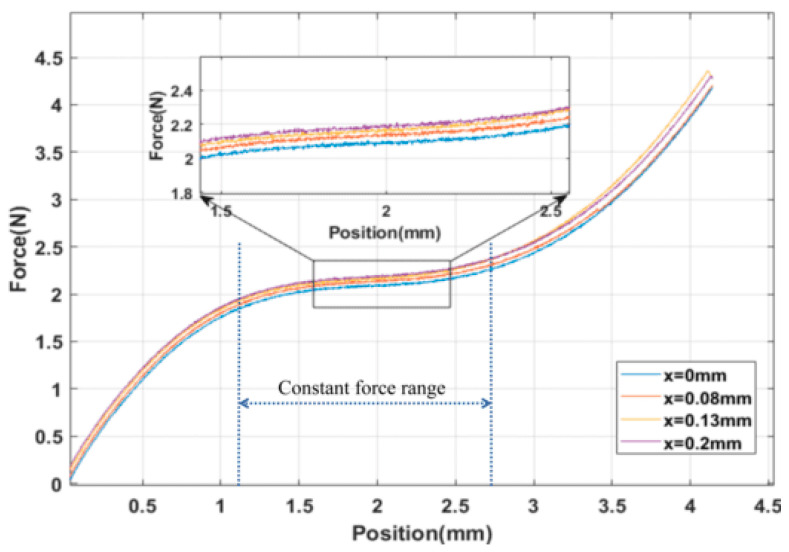
Influence of adjusting module on output of microgripper.

The test results regarding the influence of the adjusting module on the constant-force output of the microgripper are presented in Figure 16. It illustrates that the output force varies under different preload input displacements. To provide a detailed view, the force–displacement curve is locally enlarged using a displacement of 2 mm as an example. As the output displacement of the adjusting module gradually increases from 0 mm to 0.2 mm, the constant output force of the microgripper model also gradually increases from 2.05 N to 2.2 N. The adjustable constant-force output range is approximately 0.15 N. By comparing Figure 9 and Figure 16, it is evident that both the experimental and simulation results successfully demonstrate the capability of adjusting the microgripper’s constant-force output.

The results of this experiment provide confirmation that the microgripper possesses the capability to replace complex force-position controllers with a single-position controller. As demonstrated in Figure 16, it is evident that by controlling the output position of the microgripper within the constant-force stroke range of 0.855 mm to 2.565 mm, a consistent output force of approximately 2 N can be achieved. Additionally, the experiment exhibits the stepless adjustable constant-force performance of the developed microgripper. By referring to the results in Figure 16, the required preload displacement can be calculated based on the desired constant force. Subsequently, the driving voltage of the PZT can be determined using the relationship depicted in Figure 13, which outlines the correlation between the driving voltage of the PZT and the preload displacement of the adjusting module. Consequently, it becomes feasible to achieve a constant-force output of any desired values within the adjustable constant-force range.

## 5. Conclusions

In this work, an adjustable compliant constant-force microgripper has been proposed for achieving precise force control without the need for force feedback. The constant-force mechanism is automatically optimized based on finite element analysis and the MOGA method. This approach eliminates the time-consuming parameter tuning and complex model design typically required in the conventional design of CFMs while also achieving a larger constant-force stroke. To enable stepless adjustment, a PZT-driven bridge-type amplifier is employed to provide accurate preload displacement to the constant-force module. Finite element analysis and experimental verification are conducted for the adjusting module and the microgripper. The simulation and experimental results confirm that the designed microgripper successfully achieves the desired constant-force output and stepless adjustable performance. The proposed microgripper design framework offers the advantages of compact size and the ability to adjust constant-force output within a certain range. This work provides a further basis for the application and promotion of compliant constant-force structures. Future research can focus on improving the compactness of the structure, optimizing the configuration of the microgripper, expanding the adjustable range of the constant-force microgripper, and enhancing the output accuracy of constant force for better application in related fields.

## Figures and Tables

**Table 1:** Parameters of the preliminary constant-force mechanism.

	Parameter	Value	Unit
Bistable beam	$l_{b}$	25	mm
	$t_{b}$	3	mm
	$\omega_{b}$	1.1	mm
	θ	5	°
U-shape beam	$l_{f}$	20	mm
	$t_{f}$	3	mm
	$\omega_{f}$	1.1	mm

**Table 2:** Mechanical properties of ABS plastic materials.

Property	Value	Unit
Density	1040	kg/m^3^
Young’s Modulus	2.39	GPa
Poisson’s Ratio	0.399	—
Bulk Modulus	3.944	GPa
Shear Modulus	0.854	GPa
Tensile Yield Strength	41.4	MPa
Tensile Ultimate Strength	44.3	MPa

**Table 3:** Optimal structural parameter values.

Parameter	Value	Unit
t	1.8	mm
θ	5.1	°
ω	1.0	mm
$l_{f}$	16.9	mm

## Data Availability

Data are contained within the article.
